# A Novel Approach to Improve Newborn Screening for Congenital Hypothyroidism by Integrating Covariate-Adjusted Results of Different Tests into CLIR Customized Interpretive Tools

**DOI:** 10.3390/ijns7020023

**Published:** 2021-04-23

**Authors:** Alexander D. Rowe, Stephanie D. Stoway, Henrik Åhlman, Vaneet Arora, Michele Caggana, Anna Fornari, Arthur Hagar, Patricia L. Hall, Gregg C. Marquardt, Bobby J. Miller, Christopher Nixon, Andrew P. Norgan, Joseph J. Orsini, Rolf D. Pettersen, Amy L. Piazza, Neil R. Schubauer, Amy C. Smith, Hao Tang, Norma P. Tavakoli, Sainan Wei, Rolf H. Zetterström, Robert J. Currier, Lars Mørkrid, Piero Rinaldo

**Affiliations:** 1 Norwegian National Unit for Newborn Screening, Division of Paediatric and Adolescent Medicine, Oslo University Hospital, 0424 Oslo, Norway; alerow@ous-hf.no (A.D.R.); Stoway.stephanie@mayo.edu (S.D.S.); rdpetter@ous-hf.no (R.D.P.); 2 Biochemical Genetics Laboratory, Department of Laboratory Medicine and Pathology, Mayo Clinic, Rochester, MN 55905, USA; a.fornari2@campus.unimib.it (A.F.); Norgan.andrew@mayo.edu (A.P.N.); Piazza.amy@mayo.edu (A.L.P.); 3 Centre for Inherited Metabolic Diseases, Karolinska University Hospital, 17177 Solna, Sweden; henrik.ahlman@sll.se (H.Å.); rolf.zetterstrom@sll.se (R.H.Z.); 4 Division of Laboratory Services, Kentucky Department for Public Health, Frankfort, KY 40601, USA; Vaneet.Arora@ky.gov (V.A.); AmyC.Smith@ky.gov (A.C.S.); Sainan.Wei@ky.gov (S.W.); 5 Wadsworth Center, New York State Department of Health, Albany, NY 12237, USA; michele.caggana@health.ny.gov (M.C.); joseph.orsini@health.ny.gov (J.J.O.); norma.tavakoli@health.ny.gov (N.P.T.); 6 Fondazione MBBM/Ospedale San Gerardo, University of Milano-Bicocca, 20900 Monza, Italy; 7 Georgia Department of Public Health, Atlanta, GA 30303, USA; arthur.hagar@dph.ga.gov (A.H.); patricia.hall@dph.ga.gov (P.L.H.); 8 Division of Laboratory Pathology External Applications, Department of Information Technology, Mayo Clinic, Rochester, MN 55905, USA; Marquardt.gregg@mayo.edu (G.C.M.); Miller.bobby@mayo.edu (B.J.M.); Schubauer.neil@mayo.edu (N.R.S.); 9 Virginia Department of General Services, Division of Consolidated Laboratory Services, Richmond, VA 23219, USA; christopher.nixon@dgs.virginia.gov; 10 Genetic Disease Screening Program, California Department of Public Health, Richmond, CA 94804, USA; Hao.Tang@cdph.ca.gov; 11 Department of Molecular Medicine and Surgery, Karolinska Institutet, 17177 Stockholm, Sweden; 12 Department of Pediatrics, University of California, San Francisco, CA 94143, USA; Robert.Currier@ucsf.edu; 13 Department of Medical Biochemistry, Division of Laboratory Medicine, Oslo University Hospital HF, 0424 Oslo, Norway; lamo2@ous-hf.no; 14 Department of Medical Biochemistry, Institute for Clinical Medicine, University of Oslo, 0130 Oslo, Norway

**Keywords:** bioinformatics, Collaborative Laboratory Integrated Reports (CLIR), dual scatter plot, congenital hypothyroidism, covariate-adjusted reference intervals, false positives, newborn screening, single condition tool, thyroid-stimulating hormone, thyroxine

## Abstract

Newborn screening for congenital hypothyroidism remains challenging decades after broad implementation worldwide. Testing protocols are not uniform in terms of targets (TSH and/or T4) and protocols (parallel vs. sequential testing; one or two specimen collection times), and specificity (with or without collection of a second specimen) is overall poor. The purpose of this retrospective study is to investigate the potential impact of multivariate pattern recognition software (CLIR) to improve the post-analytical interpretation of screening results. Seven programs contributed reference data (N = 1,970,536) and two sets of true (TP, N = 1369 combined) and false (FP, N = 15,201) positive cases for validation and verification purposes, respectively. Data were adjusted for age at collection, birth weight, and location using polynomial regression models of the fifth degree to create three-dimensional regression surfaces. Customized Single Condition Tools and Dual Scatter Plots were created using CLIR to optimize the differential diagnosis between TP and FP cases in the validation set. Verification testing correctly identified 446/454 (98%) of the TP cases, and could have prevented 1931/5447 (35%) of the FP cases, with variable impact among locations (range 4% to 50%). CLIR tools either as made here or preferably standardized to the recommended uniform screening panel could improve performance of newborn screening for congenital hypothyroidism.

## 1. Introduction

Newborn screening (NBS) for congenital hypothyroidism (CH) has been performed globally since the 1970s, but despite broad worldwide implementation and a limited range of analytical methods, there is surprisingly little consensus around the testing protocols in place for reporting abnormal results [1,2]. With the additional uncertainty around the long-term benefits of treatment and follow-up for mild CH [3,4,5], a consensus approach to interpretation of the initial screening results has evolved to a strategy of minimizing false negative (FN) screening results [5,6]. The consequence for sites choosing to screen more broadly than consensus guidelines recommend [7] is perhaps the highest false positive (FP) rate of any NBS disorder [8], and there is a pervasive lack of standardized screening.

Currently, screening strategies for CH fall into two categories. On one end are the majority of programs which screen using a first-tier thyroid-stimulating hormone (TSH) algorithm, and on the other are those which measure thyroxine (T4) either in combination with TSH or use TSH only as a second-tier screen when T4 is below a predetermined threshold (for example the 10th percentile). Each algorithm has advantages and disadvantages, but all have a significant recall rate due to false positive results [2,9]. The false positive results obtained in newborn screening for CH are mainly due to the variability of T4 and TSH depending on time of specimen collection and prematurity. In healthy term infants, there is a TSH surge at birth stimulating T4 secretion that peaks at 24–36 h and gradually falls in the first 4 weeks after birth [10]. Due to the immaturity of the hypothalamic-pituitary-thyroid axis, preterm infants have smaller increases in serum TSH and free T4 than do term infants leading to a disproportionate number of false positive results for preterm infants who are tested by an algorithm that includes T4. In addition to time of specimen collection, birth weight, and prematurity, other factors that could influence T4 and TSH values include ethnicity, sex, maternal thyroid disease, maternal iodine status, and medication [11,12,13]. The reported incidence of CH is 1 in 2000–4000 births but has increased in recent years most likely due to lowering of TSH cut-offs, increasing survival of preterm infants, and changes in population demographics [2,14,15,16]. Lowering TSH cut-offs increases the detection of subclinical CH. However, detection and the need for treatment for babies with subclinical CH are controversial [17,18]. While there are no simple solutions to the dilemmas of CH newborn screening (i.e., selection of algorithm and cut-offs and factoring the variables involved), reducing the high incidence of preventable FP results, especially in premature newborns, is a more actionable endeavor.

Collaborative Laboratory Integrated Reports (CLIR) is a web application that maintains an interactive database of laboratory data contributed by multiple sites internationally. The development of CLIR started in 2004 as multivariate pattern recognition software to support Region 4 Stork (R4S), a performance improvement project focused on expanded newborn screening by tandem mass spectrometry [19,20,21]. Upon completion of the R4S project in 2012, the use of the software had evolved to include additional newborn screening testing scenarios [22]. The CLIR tools assist with the resolution of any condition with an available set of confirmed cases (disease ranges) and enable users to arbitrate between paired conditions (such as TP vs. FP cases) with overlapping laboratory results [20,22,23]. CLIR software enables adjustment of patient results by covariates such as birth weight and age at collection [22] and compares them to continuous moving percentiles, rather than traditional discrete reference intervals. Moving percentiles are calculated from a large body of normal data contributed by participating sites to the CLIR database and are, therefore, able to describe the dynamic pattern of physiological variation for any marker across a wide spectrum of covariates. This novel process allows users to interpret results individualized for each patient and to better recognize a true pathological finding, rather than a mere deviation from an arbitrary decision limit applied either unilaterally, or to a broad partition bin. This process offers frequent opportunities to drive down the cost of healthcare by reducing, or possibly eliminating, unnecessary patient follow-up and laboratory testing, and NBS for CH, with its disproportionate share of FP results, is a prime candidate for this approach.

We report here a retrospective study aimed at the creation of customized site-specific tools for the comparison between three alternative testing models, with a focus on the prevention of FP outcomes [21,22,23]. This study is an extension of our previous work contributing to the pursuit of newborn screening performance improvement with a focus on integrating the results of separate analytical tests performed on the same sample as a merged biochemical profile, rather than as a collection of markers to be interpreted in isolation. We describe several new features in CLIR (see Section 2.3, Section 2.4, Section 2.5 and , Section 2.6) and a significant improvement in the adjustment builder allowing for the use of two covariates. The rationale is to limit the effect of variations in, to name but a few, sample collection routines, blood availability, hematocrit, analytical methods, instrumentation, and local conditions, which lead to unnecessarily large variability when comparing sample measurements, particularly against a fixed cutoff. In summary, the underlying hypothesis of this retrospective study is that tools built with calculated ratios to unrelated markers measured by different tests but from the same sample are a more reliable alternative to single marker interpretation, since they may be proportionally influenced by the above-mentioned variations. This procedure could, thus, mitigate the variability and improve the specificity of post-analytical interpretation.

## 2. Materials and Methods

### 2.1. Analytical Methods

Routine NBS data for up to 12 markers were obtained retrospectively from the seven programs (five from the US and two from Europe) listed in the header of Table 1. For this study, we selected the primary markers of five conditions included in the Recommended Uniform Screening Panel (RUSP) [24,25] and screened for by a single marker, plus galactocerebrosidase activity (GALC, Krabbe disease). Acid α-glucosidase (GAA, Pompe disease) and C26:0-lysophosphatidylcholine (C26, X-ALD) were also included initially as markers of two other RUSP conditions but were later excluded because a significant proportion of values were missing in the Validation dataset of the only program that was measuring them during the time frame of this study. For proof of concept, citrulline (CIT), tyrosine (TYR), propionylcarnitine (C3), and palmitoylcarnitine (C16) were chosen from the larger available panel of amino acids and acylcarnitines because CIT is an amino acid that is less likely to be influenced by total parenteral nutrition [26], TYR is strongly affected by prematurity [27], and both C3 and C16 concentrations are abundant species in neonatal dried blood spot but also display a strong age-dependency [28]. Instrumentation, methods, and choices of reagents were according to local protocols; most but not all relied on Neobase non-derivatized kit for MS/MS and Genetic Screening Processor (GSP^®^) kits purchased from Perkin Elmer (Turku, Finland).

### 2.2. Reference Data 

The seven programs were selected to represent three alternative first-tier testing strategies: (1) TSH only (California, Norway, and Sweden); (2) TSH and T4 (Georgia and Kentucky); and (3) T4 followed by TSH as second-tier test (New York and Virginia). United States programs provided normal profiles (i.e., cases reported as screening negative for congenital hypothyroidism) with a collection date before 14 March 2015. This protocol complied with the section 12 provision of the Newborn Screening Saves Lives reauthorization act of 2014 that went into effect on 15 March 2015 [29], limiting research uses of non-identified results. Table 2 shows a summary of the contributions by each site and the total count of uploaded reference cases. The programs included in this study collect only one screen routinely, with local protocols for low-birth-weight infants and those in the NICU.

Each de-identified profile was expected to include four covariates: age at collection in hours, birth weight in grams, gestational age in weeks (not available for the Georgia cohort), and sex. Exclusion criteria were then applied as follows: (1) missing covariates; (2) Age > 1 yr. (8760 h); (3) Birth weight < 250 and >10,000 g; (4) Marker results shown as zero, negative values, and combined with non-numerical characters (“unsatisfactory,” “null,” “<” or “>”). These instances are listed in Table 2 as marker errors. Any of these criteria determined the exclusion of the entire case, which overall corresponded to less than 2% of the initial data. When age was listed with a value between zero and one hour, it was rounded up to one. Further removal of individual analyte values is described below. Throughout this paper, we have used local definitions for cases that were confirmed as TP. As general guidance, we consider a TP to be an infant that was identified with abnormal results by NBS and subsequently confirmed to have a targeted disorder. FP results are those infants who were identified with abnormal results by NBS and were not confirmed to have a targeted disorder, either by confirmatory testing or repeat NBS, based on local protocols.

### 2.3. Automated Removal of Reference Outliers by the Data Validation Tool

Following the removal of ineligible cases, location data were formatted separately as comma separated value (.csv) files and submitted to CLIR using an automated process called the Data Validation Tool. At first, all data were uploaded without any filtering to establish a cumulative median. To avoid interference by data already uploaded by programs worldwide to the production environment (https://clir.mayo.edu; accessed 21 April 2021), this analysis was performed in a development and testing environment inside the Mayo Clinic firewall that was free of any additional data. Next, the same files were uploaded individually, and each marker was plotted against the cumulative reference intervals. 

**Figure 1:**
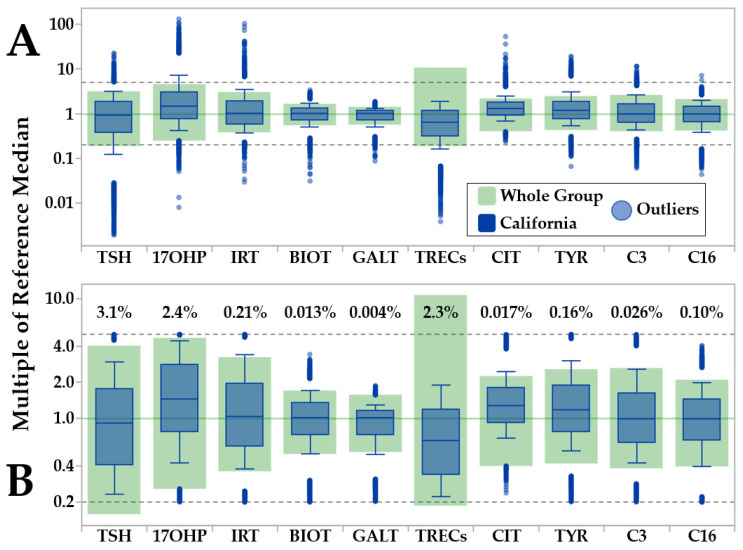
CLIR Data Validation Tool (pre- and post-outlier removal). Legend: Example of outlier removal by the CLIR Data Validation tool. Color coding is embedded in the top panel. (Panel **A**): pre-outlier removal comparison between preliminary reference ranges (based on eligible cases) and data from the largest single cohort (California, N = 533,054). (Panel **B**): post-outlier removal. Percent values above the file ranges refer to the proportion of case results that were removed for each marker.

Figure 1 shows the processing of all data from California (N = 533,054). All data above and below the 99th percentile and 1st percentile, respectively, are shown individually as outliers (blue dots). The high and low thresholds to consider a marker value to be an outlier are shown as grey dotted lines above and below the central part of the plot. The line above is equal to 5 multiples of the cumulative median, the line below is equal to 0.2 (one fifth) multiples of the cumulative median, respectively. Removal of the outliers is executed by selecting an interactive function called Outlier Removal, not shown in the figure. As expected, no profile had all values classified as outliers, so the total count of samples remained the same but counts by individual markers inevitably differ after the removal of outliers.

### 2.4. Automated Removal of Reference Outliers by the Reference Data Review Tool

**Figure 2:**
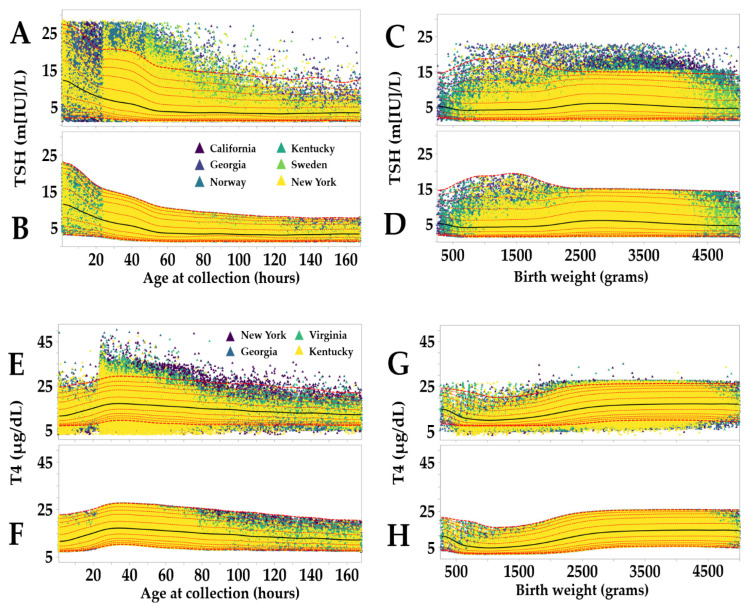
CLIR Reference Review tool (pre- and post-outlier removal by moving percentiles). Legend: Outlier removal by the CLIR Reference Data Review tool for markers TSH and T4. After the Data Validation tool, a first iteration of outlier removal (>99th percentile and <1st percentile; sorted by age panels **A** and **E**; sorted by birth weight panels **C** and **G**) was performed to transition from the uniform and inevitably flat-lined removal of outliers above and below the multiples of median (MoM) limits to a recognizable biological trend according to the selected covariate. Color coding of locations is embedded in panel **B** (TSH) and E (T4). Colors are assigned by count in descending order and are not the same for TSH and T4. (Panel **A**): Overlay of individual points by location and moving percentiles (first iteration) of TSH over one week (168 h) of age at collection in 1-h increments. Percentiles shown in all panels are: 99th percentile (thicker dotted red line), 97.5th percentile, 95th percentile, 90th percentile, 75th percentile, 50th percentile (black continuous line), 25th percentile, 10th percentile, 5th percentile, 2.5th percentile, 1st percentile (thicker dotted red line). (Panel **B**): Moving percentiles of TSH by age after second iteration of removal of values outside the peripheral percentiles. (Panel **C**): Overlay of individual points by location and moving percentiles (first iteration, performed after removal of outliers by age) of TSH over the birth weight range 250–5000 g in 25 g increments. (Panel **D**): Final moving percentiles of TSH by birth weight after the second iteration of removal of values outside the peripheral percentiles. (Panels **E**–**H**): T4 percentiles following the same process described in panels **A**,**D**.

The process outlined above is independent of covariates. To factor in age and birth weight and remove an additional layer of outliers, CLIR offers a function called Reference Data Review. By selecting a marker, a certain covariate (for the examples shown in Figure 2, the markers are TSH on the left and T4 on the right side), the covariate age (Panels A,B,E,F, range 1–168 h), the birth weight (Panels C,D,G,H, range 250–5000 g), and a display option (individual points by location, color codes are shown as inserts in Panel B,E), the distribution of the marker over the range of the covariate is displayed with the ability to overlay continuous moving percentiles.

Moving percentiles are generated by another CLIR tool called Reference Range by Covariate. It requires the selection of one marker and one continuous covariate with the option to separate data according to a categorical covariate (Male/Female; not used for TSH or T4 but applied to 17OHP and related ratios), a covariate range, and a unit of increment chosen according to data density as shown in Table 3. Increasing increments are required to avoid gaps (zero data for a given value of covariate) and enhance the smoothness of the moving percentiles. Ranges where <1% of data are reviewed by manual removal of obvious outliers based on a visual projection of the trend from the closest range with moving percentiles.

Moving percentiles are generated on demand and can be influenced by the choice of average model (simple-default, weighted, count adjusted, and a combination of weighted and count adjusted), moving average window range (3, 5, and 7 increments), and smoothing iterations (1× to 5×). In this study (see Figure 2), the choice of parameters for all markers were: (a) simple average, (b) average of 7 increments, and (c) 5 smoothing iterations.

### 2.5. Minimum-Maximum Normalization of Moving Percentiles

To overlay and compare unitless trends by covariates of different markers, the values for each increment are transformed using a normalization process described previously [10]. Briefly, this calculation transforms case scores so that the maximum value for the group is 100 and the minimum is 0 (zero). Each result is calculated by subtracting from the score the lowest of all scores, dividing it by the range of values (highest minus lowest), and multiplying by 100. This formula preserves the relative distance between values and is ideal to compare different markers. See the results section for an illustration of how it was used.

### 2.6. Ratio Explorer

Ratios to TSH and T4 with all other markers measured by at least one location were created automatically by the Ratio Explorer function, which also calculated unadjusted reference intervals. The outlier removal was limited to the primary markers, so there was no further processing by the Reference Data Review tool. The choice of denominator for individual ratios was based on the marker with the higher cumulative unadjusted median. Overall, 23 ratios were established and are shown in Supplemental Tables S2 and S3.

### 2.7. Adjustment Builder

Within this study, analytes exhibited variation across two continuous covariates, age and birth weight, as well as between locations. TSH was normalized based on the following statistical regression techniques to account for these sources of variation. Data were collected from multiple locations and then binned across a two-dimensional grid corresponding to age and birth weight. Medians and standard deviations were calculated within each bin. Polynomial regression models of the fifth degree (quintic) that incorporated values of a marker or ratio, age, and birth weight values in addition to a location factor were fit to both binned parameters to create three-dimensional percentile surfaces. Marker transformations were selected by an automated comparison between optimal Box-λ [30] and log base 10 performed by the CLIR Adjustment Manager function where an overall weighted score is calculated based on four criteria ranked from highest to lowest in this order: (a) symmetry of outlier distribution above and below the median plane; (b) total count of outliers; (c) R-squared value of the standard deviation; and (d) R-squared value of the median. A Box-Cox transformation was applied to TSH and a log base 10 transformation was applied to age and birth weight values to provide the best fit of the regression surfaces to the data. Regression outliers were identified and eliminated using a Tukey fence value of 2 multiplied by the interquartile range (IQR). The resultant regression models were used to calculate Z-scores, and the Z-score formula was applied to all reference and case data to normalize TSH values across the range of both covariates and across all locations. For T4 values, after fitting quintic polynomial models to both the median and standard deviation bins, a log base 10 transformation was applied to T4, age, and birth weight values to provide the best possible fit of both regression surfaces. Since the polynomial regression has a high order (quintic), it is essential to control the behavior and the ends of the covariate range to avoid occurrence of the Runge phenomenon [30]. Outliers were eliminated by Tukey fences and a Z-score formula was obtained and applied to all T4 values. All calculated ratios included in the study were processed in a comparable manner to account for variation across age, birth weight, and location.

### 2.8. Study Cohort

Participating locations contributed two sets of true positive (TP) and false positive (FP) cases, as resolved according to local protocols. The counts of cases are shown in Table 4. 

The Validation (training) set included cases from variable start dates between 2011 and 2013 and ended on 30 June 2014. For the reason described earlier, the Verification (testing) set covered the period 1 July 2014to 14 March 2015 with one exception: Kentucky, which ended the collection of cases on 31 December 2014. Sweden, however, contributed all TP and FP cases of the years 2017 and 2018 to the Validation and Verification group, respectively. No additional information was either provided or sought regarding confirmatory testing and clinical outcome to avoid interfering with the de-identified status of individual cases. Repeat samples are likely to be included, but no effort was made to link and compare profiles from the same case.

Cases were sorted on the basis of a single or two abnormal findings, not according to the cutoff values utilized by each program at the time of testing, but rather in comparison to the unadjusted percentiles established in this study after the final step of age and birth weight outlier removal (TSH > 99th percentile of 14.22 m[UI]/L; T4 < 1st percentile of 9.45 µg/dL). The total number of cases was 16,570, 69% of them in the Validation group. TP cases represented less than 10% of all cases in both cohorts, but there was a substantial difference in the true/false positive ratios between programs testing only for TSH (median 1.78, range 0.98–2.10) and those also using T4 either as first- or second-tier test (median 0.13, range 0.03–0.18). Unadjusted reference and disease ranges were created automatically for the markers and ratios calculated as described earlier and are shown in Supplemental Tables S1–S6. Side by side comparisons between TP and FP cases for each condition are shown in Supplemental Figures S1–S3.

### 2.9. Covariate Distribution of True and False Positive Cases

**Figure 3:**
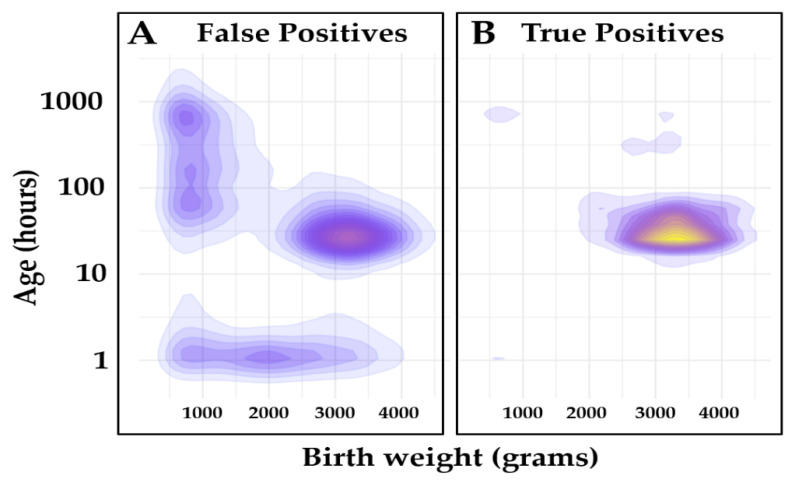
Covariate distribution of the Validation set. Legend: Covariate density plot of false positive (N = 10,556) and true positive (N = 915) cases from the Validation set. (Panel **A**): False positives; (Panel **B**): True positives. Kernel density estimation (KDE) function [31] was used to calculate probability density function of each class, and it was displayed using a contour plot. The unit values are the same as the input unit values. The scale is log10 on the *Y* axis and linear on the *X* axis of the plot. Color gradients are relative to case count.

Figure 3 shows a density plot of age at collection and birth weight of the true and false positive cases in the Validation set listed in Table 4. It clearly shows the disproportionate aggregation of false positive cases when the age at collection in newborns close to 1 h, 1 week, or <2500 g of birth weight.

### 2.10. Post Analytical Interpretive Tools: Single Condition Tools

For the purpose of building Single Condition Tools, Validation cases were sorted in six target conditions as follows: (a) CH TSH T4, true positive cases with high TSH and low T4; (b) FP TSH T4, false positive cases with high TSH and low T4; (c) CH TSH, true positive cases with high TSH (T4 either not measured or normal); (d) FP TSH, false positive cases with high TSH (T4 either not measured or normal); (e) CH T4, true positive cases with low T4 and TSH normal; and (f) FP T4, false positive cases with low T4 and TSH normal. The process to create a Single Condition Tool has been described previously [20,32]. Briefly, it consists of a sequential selection of: (a) configuration parameters (scoring and correction factor strategies); (b) location; (c) high and low markers (displayed only if provided by a given location, then chosen on the basis of a degree of overlap between reference and condition-specific disease ranges of less than 50%); (d) adjustments (standardized to age/birth weight/location with two exceptions: inclusion of sex for 17OHP and related ratios and adjustment for age/birth weight for markers unique to a location, for example GALC activity included in the New York tools); (e) marker exceptions (forced zero score when the primary marker is not abnormal), and (f) interpretation guidelines. The threshold for an informative score is set halfway between the lowest score of a case in the Validation cohort and zero. If one or more cases had a score of zero, a common occurrence with false positive conditions, the threshold was then set at a value of 1. Above the informative threshold, the likelihood of disease was stratified by quartile: <1Q (possibly), 1–3Q (likely), and >3Q (very likely). An example of the Single Condition Tool (condition CH TSH, location California) is shown in Supplemental Figure S4. Tools for false positive conditions were automatically made identical to the true positive counterparts, with the only difference of condition-specific numerical threshold of the likelihood of disease.

### 2.11. Post Analytical Interpretive Tools: Dual Scatter Plots

**Figure 4:**
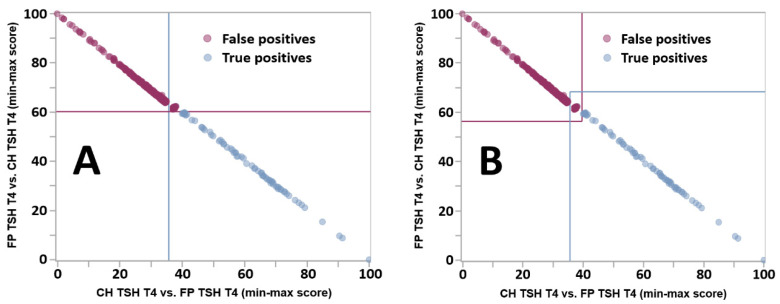
Improved definition of indeterminate cases by the Dual Scatter Plot. Legend: Dual Scatter Plot customized for location Georgia applied to the differential diagnosis between CH with high TSH and low T4 (CH TSH T4) and false positive cases with the same phenotype (FP TSH T4). The total counts of cases for true positive and false positive cases were 117 and 1360, respectively. The count of Indeterminate cases was 900, 46 (39%) true positives and 854 (63%) false positives. (Panel **A**): original design. Each plot is divided in four quadrants: Lower right: consistent with CH TSH T4 (light blue circles); Upper right: indeterminate (both conditions are possible); Upper left: consistent with FP TSH T4 (purple circles); Lower left: neither condition. (Panel **B**): new design. See text for a description of the line drawing.

Once matching pairs of tools for TP and FP with the same phenotype had been created, they were merged by an automated process into dual tools and then into a Dual Scatter Plot (DSP), an instrument of differential diagnosis previously applied successfully to the prevention of false positive outcomes [20,23]. In a DSP, the rules are different from the Single Condition Tools because the relationship to the reference range becomes irrelevant as the comparison takes place between two condition ranges. If the result falls within the range of overlap [20], there is no score modification, and therefore, no assignment to either one or the other condition. However, if the result is either below or above the area of overlap, it triggers a score modification that is proportional to the degree of separation from the range of the other condition. Figure 4 illustrates the improvement in the distribution of those cases that could not be assigned to either condition, a situation described as “Indeterminate.”

In the original version of the software [20], the classification as indeterminate referred to the entire upper right quadrant of the plot. When no cases are resolved as indeterminate, indicating a complete separation between two conditions, each line is drawn at the midpoint of the gap on each axis between values of the two conditions [32]. When overlap takes place, the orthogonal lines shown in panel A were selected as follows: the vertical line (blue) is drawn on the *X*-axis to the right of the lowest score for condition 2 (purple dots, false positives) that does not overlap with cases of condition 1 (blue dots, true positives), which is indeed the criterion used to categorize cases as indeterminate; the horizontal line (purple) on the *Y*-axis is drawn above the highest score for condition 1 that does not overlap with cases of condition 2. While the *XY* coordinates of the indeterminate quadrant could be quite variable [20], indeterminate cases are inevitably clustered tightly in the bottom left corner of the quadrant (Figure 4). In a subsequent version of the software (code version 2.16 released on 7 August 2019; current version is 2.22.01 released on 12 January 2021), the line coordinates described above become points of inflexion between two perpendicular lines (Figure 3B), together creating a central rectangle that contains all indeterminate cases; the bottom left and top right partial quadrants become neutral to the resolution of cases. The ability to completely segregate the zone with indeterminate cases is needed to resolve the distribution of cases when two perpendicular lines are not capable of separate the three groups, an outcome that is required for the zoom function described below.

### 2.12. Zoom Function of the Dual Scatter Plot

A novel feature of this plot is introduced here for the first time, and is called Zoom Plot. This function follows the same principles of the parent Dual Scatter Plot, but limits the comparison of disease ranges only to the Validation cases included in the indeterminate zone. As such, range separation is found in much smaller numerical differences that would not be recognized within the full disease range. See the results section for a visual representation of the impact of this functionality.

### 2.13. Dual Scatter Plot Runner

Verification cases were sorted into separate files according to the six conditions described above and uploaded individually to the Dual Scatter Plot Runner. This tool operates sequentially a Single Condition Tool and the associated Dual Scatter Plot. After a location-specific tool is selected, users need to select the source of reference range values (either cumulative or location; default is cumulative), filter (one or both conditions need to have either an informative score or a non-zero score; default setting is that the true positive tool must have an informative score), guidelines (as defined in the tool), and finally whether to apply the zoom sorting of indeterminate cases (default is yes). The final step is selecting the file to be processed; the computation time for the largest file of the Verification set (N = 1140) was approximately 10 s, irrespective of hardware and browser.

## 3. Results

### 3.1. Minimum-Maximum Normalization of Moving Percentiles

**Figure 5:**
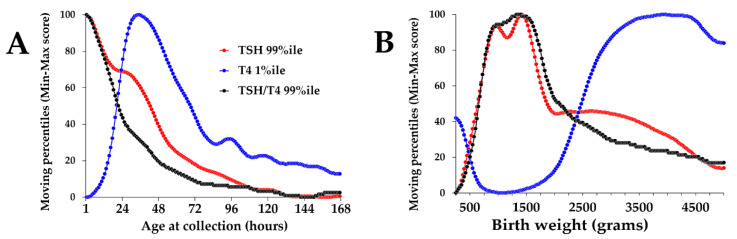
Normalized moving percentiles of TSH, T4, and the TSH/T4 ratio by age and birth weight. Legend: (Panel **A**): Normalized moving percentiles by age, range 1–168 h (1 week). Color coding of markers is embedded in the plot. (Panel **B**): Moving percentiles by birth weight, range 250–5000 g. First occurrence of maximum value: TSH: age 1 h and birth weight 1425 g; T4: age 34 h and birth weight 3825 g; TSH/T4 ratio: age 1 h and birth weight 1325 g.

Figure 5 is an objective illustration of why selection of static and/or binned cutoff values is prone to excessive approximation when applied to newborn screening for congenital hypothyroidism. After normalization of the moving percentiles by min-max score of more than 1 million data points, it becomes evident that even small increments of covariate (1 h up to 1 week of age and 25 g up to 5000 g) result in noticeable variations of the peripheral percentiles, meaning that a given result could be misinterpreted, especially in samples collected before 24 h of age and in premature cases born less than 2500 g of weight. As expected, the two primary markers and consequently the calculated ratio between them behave very differently, trending in opposite directions (TSH declining and T4 increasing) in the first 48 h and especially across the entire spectrum of birth weight in premature newborns.

Hence, the alternative we propose is the creation and reliance on simultaneous, multiple covariate-adjusted reference intervals.

### 3.2. Reference Intervals Adjusted for Age, Birth, Weight and Location

**Figure 6:**
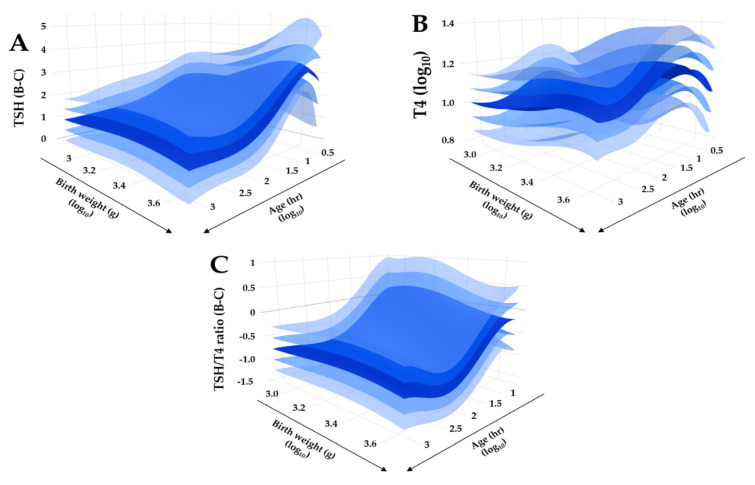
Regression quality control plot of covariate adjusted TSH, T4, and TSH/T4 ratio. Legend: Bi-dimensional plots in a tri-dimensional space of adjusted reference intervals. (Panel **A**): TSH, Box-Cox transformation; (Panel **B**): T4, log10 transformation; (Panel **C**): TSH/T4 ratio, Box-Cox transformation. Primary covariate is age (right lower axis), secondary covariate is birth weight (left lower axis). The dark blue surface represents the median, lighter blue surfaces represent one and two standard deviations above and below the median, respectively.

Figure 6 shows visual representations of the adjustment calculated for TSH, T4, and the TSH/T4 ratio, respectively. The creation of these plots is automated by a CLIR tool called Adjustment Manager that can perform a transformation comparison and scoring for batches of selected markers and generate an interactive report where an authorized user with proper statistical expertise selects and saves the equation with the best fit.

**Figure 7:**
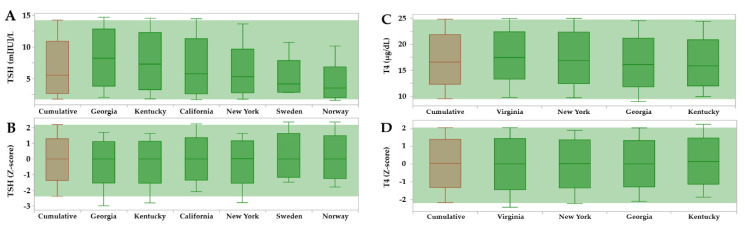
Reference Range Comparison of TSH and T4 reference ranges in dried blood spots by contributing sites. Legend: The horizontal green band overlays the cumulative peripheral percentiles (brown) on top of the individual sites (dark green). Locations are not aligned vertically because they were sorted left to right in descending order of the median separately in each panel. (Panel **A**): unadjusted TSH ranges; (Panel **B**): TSH ranges after adjustment for age (hours), birth weight (grams) and location, expressed as Z-scores; (Panel **C**): unadjusted T4 ranges; (Panel **D**): T4 ranges after adjustment for age (hours), birth weight (grams), and location, expressed as Z-scores.

Figure 7 shows a comparison of unadjusted vs. adjusted reference intervals by individual location using the CLIR productivity tool called Reference Range Comparison. The best example of improved consistency and comparability is found for TSH, where there was a 2.3-fold difference between the location with the highest median (Georgia, 8.23 m[IU]/L) and the one with the lowest (Norway, 3.52 m[IU]/L). Based on differences of time of collection (Georgia median age at collection 28.7 h, Norway 54.4 h), the trends illustrated in Figure 6 support the argument that such difference should be expected. Yet, after normalization by an adjustment that included harmonization by location, the difference at the median level was eliminated almost completely. Another notable example (see Supplemental Figure S5) was the harmonization of TREC ranges between California and New York, a different situation that likely reflects known methodological differences [33,34].

### 3.3. Dual Scatter Plot Analysis

**Figure 8:**
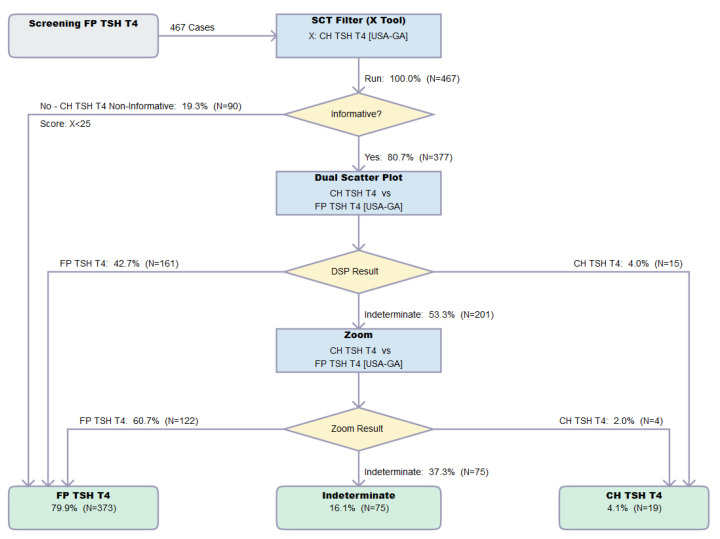
Result flow of the Dual Scatter Plot Runner. Legend: Resolution by the CLIR Dual Scatter Plot Runner of 467 FP cases with elevated TSH and low T4 from location Georgia (USA-GA). Single Condition Tool is CH TSH T4 version 026 created 1 February 2021, Dual Scatter Plot CH TSH T4 vs. FP TSH T4 version 027 created 1 February 2021. Image is shown unedited as created automatically by the software. Color coding as follows: Grey, start; Blue, process; Yellow: decision; Green, totals.

**Figure 9:**
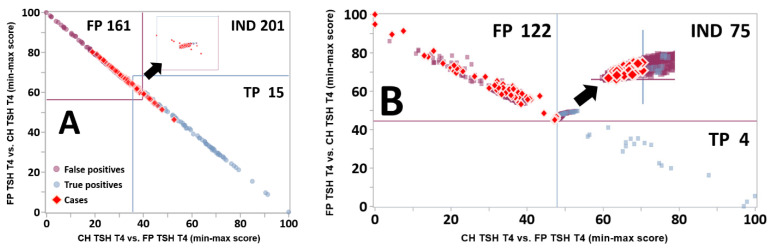
Resolution of Indeterminate cases by the Zoom Plot. Legend: (Panel **A**): Same Dual Scatter Plot for location Georgia as shown in Figure 4A, overlaid with red diamonds representing individual false positive cases from the Verification cohort (location Georgia, condition FP TSH T4, N = 467). The insert in the top right quadrant is a magnification of the Indeterminate zone. Quadrants are defined as in the legend of Figure 4. (Panel **B**): 900 Indeterminate cases (see Figure 4) from the Validation cohort overlaid with the 201 Indeterminate cases from Panel A. The insert in the top right quadrant is a magnification of the intersection of the lines.

Figure 8 shows an example of the output of the Dual Scatter Plot Runner after uploading a file from the Verification cohort. The uploaded file consisted of 467 false positive cases from Georgia with both high TSH and low T4, and not surprisingly, all cases were informative for the Single Condition Tool. However, 373 of them (80%) were resolved as false positives sequentially by the Dual Scatter Plot (see Figure 4B) and Zoom Plot (Figure 9).

### 3.4. Cumulative Outcome of the Analysis of Verification Set

Table 5 summarizes the resolution by Dual Scatter Plot Runner of true and false positive cases by location in the Verification dataset. Overall, 2% of the true positive cases were resolved incorrectly, and 36% (range 17–50% by location) of false positive cases could have been resolved properly as true negatives.

The demographic characteristics and screening results of potential false negative screens are summarized in Table 6.

It is important to note that per local protocols, each of these infants would have been required to have additional screenings performed, as none of these screenings individually would meet the requirements for a satisfactory test, based on age at collection and birth weight. Six of eight screenings had a collection time of one hour and would have been required to have another screen collected after 24 h of life. The remaining two were collected close to one month of age. Six of these cases had a birth weight <1500 g. One was missed by the Single Condition Tool, three failed to be recognized by the Dual Scatter Plot and the other four by the Zoom tool.

### 3.5. Impact of the Zoom Function toward the Resolution of FP Cases

From the perspective of interpretation of an individual case, an outcome of Indeterminate is an abnormal result that would trigger further evaluation. After the Dual Scatter Plot, 201 FP TSH T4 cases from location Georgia were classified as Indeterminate, but the Zoom Plot shown in Figure 9 resolved correctly as false positives an additional 122 of them (Panel B).

## 4. Discussion

Newborn screening for CH relies almost entirely on the determination of the concentration of TSH and/or T4 in dried blood spots. In rare cases, programs also measure thyroid binding globulin (TBG) [35] or free T4 [36], but no program measuring TBG or free T4 for screening purposes was included in this comparative study. As shown in Supplemental Figures S1–S3, TSH is a sensitive marker, and even if FP cases also show an elevated condition range, they are clearly separated from the range observed in affected cases, a difference that is exactly what CLIR tools are meant to recognize to improve specificity. This observation is true for both CH TP cases (Supplemental Figure S1) and even more pronounced in CH TSH T4 (Supplemental Figure S3, top panel; see also TSH disease percentiles in Supplemental Tables S4–S6, respectively). On the other hand, the T4 ranges of CH T4 and FP T4 cases are essentially the same (Supplemental Figure S2), suggesting that a combination of low T4 and normal TSH (“OR” algorithm) is a problematic interpretation strategy to follow and is likely the root cause behind the high number of false positives encountered by programs using T4 as either first-tier or second-tier screening. The reliability of isolated low T4 values should be reconsidered, but it is premature to suggest its outright elimination from testing protocols, as the TSH/T4 ratio, even when T4 is normal, is very informative and discriminative between TP and FP cases. When measuring both TSH and T4, further evaluation is warranted when both markers are abnormal or only TSH (plus the TSH/T4 ratio) is informative. The strategy of T4 first-tier screening followed by TSH as second-tier test could still be viable, but the TSH result should be the decision point with full overriding control of the initial T4 result [37]. An exception could be considered for programs that specifically aim to report newborns with central CH, a disorder characterized by normal TSH levels but abnormally low T4 levels. In case financial resources are allocated to screen for central CH, a presumably effective approach could be adding the measurement of total or free thyroxine to TSH.

The performance improvement hypothesis of this retrospective study is based on two premises: first, the calculation of ratios between primary CH markers and others that are routinely measured to screen for unrelated conditions creates an informative multiplex profile that could lead to the recognition of differences in the degree of overlap between analyte ranges of TP and FP cases. A higher degree of separation for a given ratio could be found unexpectedly, such as the ratios of TSH to Biotinidase activity and to Galactose-1-phosphate uridyl transferase activity (both expressed as a percentage of the daily median) as measured in Sweden (Supplemental Figure S1). Second, the physiological trends of markers of thyroid function in the neonatal period are so dynamic and fast-changing that reliance on static cutoffs, with or without broad binning for age and birth weight, is destined to lead to a frequently incorrect interpretation and unnecessary follow-up testing, especially in extremely low birth weight premature newborns, as long as current practices of collecting a dried blood spot sample immediately after birth are not revisited [38].

CLIR has been proven to achieve performance improvements for multiplex panels measured by tandem mass spectrometry for inborn errors of amino acid, organic acid, and fatty acid metabolism [20,21] and lysosomal disorders [22]. The validation of a second-tier test for Pompe disease [23] was the first instance of finding clinical utility by merging the results from two different analytical tests, the lysosomal and peroxisomal 10-plex panel [39] and a creatine disorder panel [30]. The information technology infrastructure needed to merge results of different tests is complex, but overall manageable by a variety of approaches, with a greater obstacle to be found in automated matching of analytical results with covariate information, often stored in a different system. Once such a goal is broadly recognized to add value to the quality and performance of newborn screening, it is likely that seamless solutions will become routinely available from instrument and/or reagent manufactures once they have reached a business decision to make it available to their customers.

In this study, the lack of uniformity of other markers available in addition to the three models of testing for primary CH markers was pervasive. Only 17OHP, two amino acids, and two acylcarnitines were available from all sites, and the latter were measured by either one of two different MS/MS methods (Table 1). This situation, however, was turned into an opportunity to showcase the flexibility of CLIR and to evaluate whether larger profiles could perform better in terms of FP prevention. Anecdotally, the location with the smallest available marker set also had the lowest percent improvement and was the only program that did not have at least one more ratio in its customized panel beyond the five common markers. The prevention of FP was split on average 2:2:1 between the Single Condition Tools (cases with a non-informative score, likely to be a direct effect of the adjustment for covariates), the traditional Dual Scatter Plot, and the novel function Zoom Plot. The contribution of the new feature was as high as 83% of the preventable FP cases (California, 33 of 40) and as low as none (Kentucky, 0 of 5) (Table 5). Further studies with different testing scenarios are needed to confirm the full clinical utility of this new function.

The FN screenings in this study are a concern, but they highlight the difficulty associated with interpreting NBS results at the extremes of covariate ranges because of: (a) minimal if not missing altogether reference data with comparably rare combinations of covariates, causing unforeseen extrapolations in the creation of the adjustment. Based on their demographics, each infant who had a missed screen would have had either subsequent or previous screenings. Considering Georgia’s protocol for repeat screenings and follow-up testing, subsequent normal screenings for CH would have resulted in the case being resolved as normal for a child in the NICU. While specific case level data could not be reviewed due to the deidentified nature of the study, it is extremely unlikely that these missed screenings would result in missed diagnoses. After the completion of this study, Georgia undertook a retrospective review of CH screening data to adjust cutoffs, with care taken not to miss any cases. At 1 h of age, the TSH level now needs to be >100 uIU/mL to be abnormal. As these cases were all from a similar time period, this is additional supporting evidence that these infants would have been correctly identified and referred for treatment if CLIR had been utilized. This scenario could be an underlying cause of why TSH values in the 50–60 range (Table 6) were not interpreted correctly; (b) a possible bias within the Dual Scatter Plot algorithm that might favor under certain circumstances the assignment of a case to the condition in which similar covariate values are far more common (see Figure 3). Overall, it appears from this study that performing newborn screening for CH immediately after birth in a premature/sick newborn is prone to a variety of biological and analytical artifacts that may result in harm greater than any benefit of early identification, also considering that CH might not be considered a time-critical condition [40]. Protocols have been developed for screening in NICUs to minimize missed cases; however, it may be reasonable to reevaluate the timing of collection with a goal of overall improved screening performance (reduce FP and FN results), and less interference in the provision of critical care.

No attempt was made to question the outcome classification by the contributing sites. An exception was a case from Norway with a TSH value of 4.4 m[IU]/L that was initially included in the Validation TP group, even if it was known to the program as a confirmed false negative event. There were a few additional limitations of this study that deserve to be mentioned as opportunities for improvement and as a source of learned lessons guiding future prospective studies. First, there was no correlation to actual prevalence, sensitivity, and specificity as sample exclusion criteria were applied inconsistently before submission, such as removal of any abnormal result for other conditions vs. only of cases with abnormal results for thyroid markers, further compounded by the post-submission criteria driven by a lack of data completeness (Table 2). Second, there was no objective way to compare programs to the others, as all had different panels, so each location was evaluated separately with the most basic outcome of percentage of potentially missed true positives and preventable false positives. There was no effort to exclude birth weight in older patients, because overall, they represented only a very small proportion of the study population (<1%, Table 3). Although not intrinsic to this post-analytical study, it was challenging to rationalize how cases resolved as false positives could have markedly elevated TSH values, well above the expected physiological response [41]. Finally, it could have been helpful to link repeat samples to the initial samples and to integrate the resolution by the tools with a longitudinal and integrated assessment of adjusted data. An unresolved issue is up to what age it is still relevant to use the original birth weight in the regression. Adjustment for age and location only is a viable option, but it was not included within the scope of this study, since it does not apply to most samples.

In 2018, the concept of using CLIR to build a recommended uniform screening tool (RUST) was introduced at the ISNS conference in Bratislava [42]. If even a single numerical marker is chosen for each condition (or groups having overlapping phenotypes) included in the uniform panel [24,25], screening for CH using either TSH, T4, or both could be integrated with as many as 22 (or 45) calculated ratios without any additional analytical effort. Further customization is certainly possible by individual programs using the CLIR Tool Editor and includes the options to consider other commonly measured markers to calculated ratios (tyrosine, valine, acetylcarnitine, and palmitoylcarnitine to name just a few) and also to exclude low intensity markers (argininosuccinic acid, succinylacetone, and any of the long chain hydroxyl acylcarnitine species). This universal panel is not limited to CH and could be readily applied to any other RUSP condition currently screened for by a single marker. The successful application of CLIR to the interpretation of very heterogeneous combinations of markers screening for a single disorder also highlights a major strength of the CLIR approach. While many advanced machine learning tools can be trained on high-dimensional data, they are often dependent upon every input variable in order to generate an interpretation. The realities of laboratory screening mean that complete data are not always available at any given point. Being able to customize tools to the data available in variable circumstances is a fundamental property of the CLIR design, and one that gives a level of robustness in real-world usage which is highly sought after. Based on the preliminary evidence obtained by this study, it is highly likely that such granularity of biochemical fingerprinting could lead to performance improvement and clinical utility consistent with the concept of precision newborn screening based upon near-zero FP rates [22].

## Figures and Tables

**Table 1:** First tier markers contributed by participating locations.

Marker	Unit	California	Norway	Sweden	Georgia	Kentucky	New York	Virginia
TSH	M[UI]/L	+	+ ^1^	+	+	+	- ^2^	- ^2^
T4	µg/dL	-	-	-	+	+	+	+
IRT	µg/dL	+	-	-	+	+	+	+
17OHP	ng/mL	+	+ ^3^	+	+	+	+	+
C3	nmol/mL	+ ^4^	+ ^4^	+ ^4^	+ ^4^	+ ^5^	+ ^5^	+ ^5^
C16	nmol/ml	+ ^4^	+ ^4^	+ ^4^	+ ^4^	+ ^5^	+ ^5^	+ ^5^
CIT	nmol/mL	+ ^4^	+ ^4^	+ ^4^	+ ^4^	+ ^5^	+ ^5,6^	+ ^5^
TYR	nmol/mL	+ ^4^	+ ^4^	+ ^4^	+ ^4^	+ ^5^	+ ^5,6^	+ ^5^
BIOT	ERU	+	+ ^7^	+ ^8^	+	- ^9^	- ^9^	- ^9^
GALT	U/g[Hb]	+	-	+	+	- ^9^	- ^9^	- ^9^
TRECS	copies/µL	+	-	-	-	-	+	-
GALC	nmol/mL/hr	-	-	-	-	-	+	-
Measured in this study		10	8	8	10	8	9	7

Legend: Testing by US programs was performed before 15 March 2015. (^1^) results converted from whole blood to plasma by applying a ×2.2 conversion factor; (^2^) performed as second-tier test; (^3^) results converted from nmol/L in whole blood to ng/mL in plasma by applying sequentially the conversion factors ×0.3304611 and ×2.2; (^4^) underivatized MS/MS method; (^5^) derivatized method MS/MS method; (^6^) results converted from mg/dL to nmol/mL by applying the conversion factors ×57.1 (citrulline) and ×55.2 (tyrosine); (^7^) fluorometry assay, results expressed as nmol/mL/min then converted to ERU by applying a ×0.2 conversion factor; (^8^) fluorometry assay, results expressed as the percentage of the daily median; (^9^) measured but with qualitative assay (positive/negative). Abbreviations (in alphabetical order): 17OHP, 17-hydroxy progesterone; BIOT, Biotinidase activity; C3, propionylcarnitine; C16, palmitoylcarnitine; CIT, citrulline; ERU, enzyme response unit; GALC, galactocerebrosidase activity; GALT, galactose-1-phosphate uridyl transferase activity; IRT, immunoreactive trypsinogen; T4, total thyroxine; TRECS, T-cell receptor excision circles; TSH, thyroid-stimulating hormone; TYR, tyrosine.

**Table 2:** Summary of reference data at the time of submission and after exclusion criteria.

	California	Norway	Sweden	Georgia	Kentucky	New York	Virginia	Total
Samples submitted	537,225	223,168	90,021	272,832	232,017	389,109	226,164	1,970,536
Covariate errors	4126	1093	−	6787	5164	7173	3150	27,493
Marker errors	45	259	−	78	7345	2508	35	10,270
Samples excluded	4171	1352	−	6865	12,509	9681	3185	37,763
% excluded	0.8%	0.6%	0.0%	2.5%	5.4%	2.5%	1.4%	1.9%
Samples uploaded	533,054	221,816	90,021	265,967	219,508	379,428	222,979	1,932,773

**Table 3:** Selection of unit increment by data density for the establishment of moving percentiles.

Continuous Covariate	Unit of Measure	Covariate Interval	End of Interval	Proportion of Data (%) ^a^	Unit of Increment
Age at collection	hours	1–168	1 week	97.70%	1
		169–552	1 month	1.48%	6
		553–4380	6 months	0.80%	24
		4381–8760	1 year	0.01%	n/a
Birth weight	grams	250–5000	n/a	99.86%	25
		5001–10,000	n/a	0.14%	n/a

Legend: ^a^ calculated for the marker propionylcarnitine (C3) as it is included in the dataset of all seven locations (see Table 1). The total count of C3 values after outlier removal by the Validation tool is N = 1,846,537. n/a, percentiles not calculated.

**Table 4:** Classification and distribution of true and false positive cases in the Validation and Verification datasets after application of the same exclusion criteria applied to reference data.

	Abnormal Markers												
	TSH H + T4 L		TSH H		T4 L		Total Counts by Location						
Validation	TP	FP	TP	FP	TP	FP	TP	% ^a^	FP	% ^a^	All	% ^a^	T/F Ratio
California	−	−	162	92	−	−	162	18%	92	0.90%	254	2%	1.76
Norway	−	−	47	48	−	−	47	5%	48	0.50%	95	0.80%	0.98
Sweden	−	−	65	31	−	−	65	7%	31	0.30%	96	0.80%	2.1
Georgia	122	1549	98	2635	39	3676	259	28%	7860	74%	8119	71%	0.03
Kentucky	72	49	47	668	9	232	128	14%	949	9%	1077	9%	0.13
New York	113	119	43	162	31	747	187	20%	1028	10%	1215	11%	0.18
Virginia	46	187	12	86	9	275	67	7%	548	5%	615	5%	0.12
Total	353	1904	474	3722	88	4930	915	8%	10,556	92%	11,471		
Verification													
California	−	−	143	82	−	−	143	31%	82	1.80%	225	4%	1.74
Norway	−	−	18	31	−	−	18	4%	31	0.70%	49	1%	0.58
Sweden	−	−	60	41	−	−	60	13%	41	0.90%	101	2%	1.46
Georgia	30	467	34	996	24	803	88	19%	2266	49%	2354	46%	0.04
Kentucky	10	4	8	52	2	71	20	4%	127	3%	147	3%	0.16
New York	46	119	37	161	12	377	95	21%	657	14%	752	15%	0.14
Virginia	25	179	3	122	2	1140	30	7%	1441	31%	1471	29%	0.02
Total	111	769	303	1485	40	2391	454	9%	4645	91%	5099		

Legend: Counts represent cases after application of exclusion criteria (see text). H, high (TSH > 14.22 m[IU]/L, 99th percentile of this study); L, low (T4 < 9.45 µg/dL, 1st percentile of this study); FP, false positive cases; TP, true positive cases. (^a^) Percentage of the total number of cases by location and category in the dataset.

**Table 5:** Resolution of cases by location in the Verification set.

	California	Norway	Sweden	Georgia	Kentucky	New York	Virginia	Totals	
First tier screening	TSH	TSH	TSH	TSH + T4	TSH + T4	T4	T4		
Second tier test						TSH	TSH		
Other markers (ratios)	9	7	8	8	6	8	6	52	
Single condition tools (SCT)	2	2	2	6	6	6	6	30	
Dual scatter plots (DSP)	1	1	1	3	3	3	3	15	
True positive cases	143	18	60	88	20	95	30	454	
Cases resolved as FP by SCT	-	-	-	-	-	-	-	0	
Cases resolved as FP by DSP	-	-	-	2	-	2	-	4	
Cases resolved as FP by Zoom	-	-	-	4	-	-	-	4	
Screens resolved as FP by CLIR	0	0	0	6	0	2	0	8	
%	0%	0%	0%	7%	0%	2%	0%	2%	
False positive cases	82	31	41	2732	127	657	1777	5447	
Cases resolved as FP by SCT	4	6	-	637	3	17	107	774	(40%)
Cases resolved as FP by DSP	3	-	5	489	2	133	180	812	(42%)
Cases resolved as FP by Zoom	33	3	8	229	-	55	17	345	(18%)
Screens resolved as FP by CLIR	40	9	13	1355	5	205	304	1931	
%	49%	29%	32%	50%	4%	31%	17%	35%	

Legend: TN, true negative (normal screening).

**Table 6:** True positive screening results resolved as false positive by CLIR tools.

Case	Site	Tool	Age (Hours)	Birth Weight (Grams)	Gest. Age (Weeks)	Sex	TSH (m[IU]/L	T4 (µg/dL)	Resolution by SCT	Resolution by DSP	Resolution by Zoom
Case 01	GA	TSH T4	1	1474	n/a	Male	*54*	*2.1*	Informative	Indeterminate	FP
Case 02	GA	TSH T4	1	911	n/a	Female	*53*	*4.1*	Informative	Indeterminate	FP
Case 03	GA	TSH T4	1	2535	n/a	Male	*51*	*6.0*	Informative	Indeterminate	FP
Case 04	GA	TSH T4	715	540	n/a	Male	*22*	*1.8*	Informative	Indeterminate	FP
Case 05	GA	T4	659	669	n/a	Male	8	*4.8*	Informative	FP	-
Case 06	GA	T4	1	437	n/a	Female	13	*4.6*	NI	-	-
Case 07	NY	TSH T4	1	3010	39	Male	*23*	*4.6*	Informative	FP	-
Case 08	NY	TSH T4	1	515	30.1	Male	*34*	*5.3*	Informative	FP	-

Legend: n/a, not available; NI, not informative; FP, false positive; -, not applicable. Values shown in italic and underscored are abnormal (TSH > 14.22 m[IU]/L; T4 < 9.45 µg/dL).

## Supplementary Materials

The following are available online at https://www.mdpi.com/article/10.3390/ijns7020023/s1, Table S1: Unadjusted reference percentiles of measured markers, Table S2: Unadjusted reference percentiles of calculated TSH ratios, Table S3: Unadjusted reference percentiles of calculated T4 ratios, Table S4: Unadjusted extended percentiles of disease ranges for conditions CH TSH and FP TSH, Table S5: Unadjusted extended percentiles of disease ranges for conditions CH T4 and FP T4, Table S6: Unadjusted extended percentiles of disease ranges for conditions CH TSH T4 and FP TSH T4, Figure S1: Plot by Multiple Conditions comparing disease ranges of conditions CH TSH and FP TSH, Figure S2: Plot by Multiple Conditions comparing disease ranges of conditions CH T4 and FP T4, Figure S3: Plot by Multiple Conditions comparing disease ranges of conditions CH TSH T4 and FP TSH T4, Figure S4: Single Condition Tool for condition CH TSH for location California, Figure S5: Reference range comparison of unadjusted and adjusted values for marker TRECs.

## Abbreviations

17OHP: 17-hydroxy progesterone, BIOT: Biotinidase, C16: palmitoylcarnitine, C3: propionylcarnitine, CH: congenital hypothyroidism, CIT: citrulline, CLIR: Collaborative Laboratory Integrated Reports, DSP, Dual Scatter Plot, FN: false negative, FP: false positive, GAA: Acid α-glucosidase, GALC: galactocerebrosidase, GALT: galactose-1-phosphate uridyl transferase activity, IQR: interquartile range, IRT: immunoreactive trypsinogen, ISNS: International Society for Neonatal Screening, KDE: Kernel density estimation, MS/MS: tandem mass spectrometry, NBS: newborn screening, NICU: neonatal intensive care unit, R4S: Region 4 Stork, RUSP, recommended uniform screening panel, SCT: Single Condition Tool, T4: thyroxine, TRECS: T-cell receptor excision circles, TP: true positive, TSH: thyroid-stimulating hormone, TYR: tyrosine.
