# Supplementary material for: A Novel Approach to Improve Newborn Screening for Congenital Hypothyroidism by Integrating Covariate-Adjusted Results of Different Tests into CLIR Customized Interpretive Tools
Source: Int J Neonatal Screen. 2021 Apr 23;7(2):23. doi: 10.3390/ijns7020023 (PMC8167643; doi:10.3390/ijns7020023)
Supplement: Supplementary file 1 [file IJNS-07-00023-s001.zip › IJNS-1126325-supplementary.pdf]

International Journal of
*Neonatal Screening*

MDPI

*Supplemental material*

# Enhanced performance of newborn screening for congenital hypothyroidism by integrating covariate-adjusted results of different tests into CLIR customized interpretive tools

**Alexander D. Rowe, Stephanie D. Stoway, et al**

**Table S1.** Unadjusted reference percentiles of measured markers

| Marker | Unit | 1%ile | 10%ile | 50%ile | 90%ile | 99%ile |
|---|---|---|---|---|---|---|
| TRECs | copies/µL | 99 | 157 | 382 | 1869 | 3111 |
| GALT | U/g[Hb] | 152 | 187 | 262 | 309 | 356 |
| GALT % | % | 65 | 80 | 101 | 116 | 126 |
| BIOT F% | % | 70 | 81 | 100 | 122 | 137 |
| Tyr | nmol/mL | 40 | 53 | 82 | 130 | 174 |
| BIOT A | ERU | 27 | 34 | 47 | 61 | 71 |
| IRT | µg/dL | 9.8 | 13 | 22 | 39 | 56 |
| T4 | µg/dL | 9.4 | 12 | 17 | 22 | 25 |
| Cit | nmol/mL | 6.3 | 8.4 | 13 | 21 | 26 |
| 17OHP | ng/dL | 2.4 | 3.7 | 7.3 | 15 | 22 |
| TSH | m[UI]/L | 1.8 | 2.7 | 5.5 | 11.0 | 14 |
| GALC | nmol/mL/hr | 1.4 | 2.1 | 4.0 | 7.5 | 11 |
| C16 | nmol/mL | 1.4 | 1.9 | 2.9 | 4.3 | 5.3 |
| C3 | nmol/mL | 0.83 | 1.1 | 1.8 | 2.8 | 3.8 |

Legend: Markers are sorted in descending order of the median. Values are shown to include at least two significant figures. Abbreviations (in alphabetical order): 17OHP, 17-hydroxy progesterone; BIOT, Biotinidase activity; C3, propionylcarnitine; CIT, citrulline; C16, palmitoylcarnitine; ERU, enzyme response unit; GALC, galactocerebrosidase activity; GALT, galactose-1-phosphate uridyl transferase activity; IRT, immunoreactive trypsinogen; T4, total thyroxine; TRECS, T-cell receptor excision circles; TSH, thyroid stimulating hormone; TYR, tyrosine.

**Table S2.** Unadjusted reference percentiles of calculated **TSH** ratios.

| Ratio | 1%ile | 10%ile | 50%ile | 90%ile | 99%ile |
|---|---|---|---|---|---|
| 17OHP/TSH | 0.350 | 0.600 | 1.360 | 3.240 | 6.230 |
| GALC/TSH | 0.150 | 0.280 | 0.680 | 1.810 | 4.100 |
| C16/TSH | 0.140 | 0.230 | 0.510 | 1.230 | 2.100 |
| TSH/T4 | 0.130 | 0.240 | 0.490 | 0.820 | 1.140 |
| C3/TSH | 0.098 | 0.170 | 0.370 | 0.820 | 1.320 |
| TSH/Cit | 0.083 | 0.140 | 0.310 | 0.740 | 1.350 |
| TSH/IRT | 0.056 | 0.110 | 0.260 | 0.590 | 1.000 |
| TSH/BIOT A | 0.032 | 0.054 | 0.120 | 0.250 | 0.380 |
| TSH/Tyr | 0.016 | 0.028 | 0.064 | 0.140 | 0.240 |
| TSH/GALT % | 0.023 | 0.027 | 0.043 | 0.081 | 0.120 |
| TSH/BIOT F% | 0.022 | 0.027 | 0.042 | 0.080 | 0.120 |
| TSH/GALT | 0.007 | 0.011 | 0.024 | 0.046 | 0.070 |
| TSH/TRECs | 0.002 | 0.007 | 0.019 | 0.047 | 0.088 |

Legend: Ratios are sorted in descending order of the median. Values are shown to include three decimal figures. Abbreviations as listed in the legend of supplemental Table 1.

**Table S3.** Unadjusted reference percentiles of calculated **T4** ratios.

| Ratio | 1%ile | 10%ile | 50%ile | 90%ile | 99%ile |
|---|---|---|---|---|---|
| T4/IRT | 0.260 | 0.400 | 0.740 | 1.280 | 1.840 |
| Cit/T4 | 0.310 | 0.430 | 0.700 | 1.210 | 2.000 |
| TSH/T4 | 0.130 | 0.240 | 0.490 | 0.820 | 1.140 |
| 17OHP/T4 | 0.150 | 0.230 | 0.430 | 0.840 | 1.540 |
| T4/BIOT A | 0.160 | 0.250 | 0.360 | 0.520 | 0.700 |
| GALC/T4 | 0.077 | 0.120 | 0.240 | 0.460 | 0.790 |
| T4/Tyr | 0.077 | 0.120 | 0.210 | 0.360 | 0.500 |
| C16/T4 | 0.077 | 0.110 | 0.170 | 0.270 | 0.370 |
| C3/T4 | 0.045 | 0.064 | 0.110 | 0.180 | 0.270 |
| T4/GALT | 0.033 | 0.045 | 0.066 | 0.098 | 0.130 |
| T4/TRECs | 0.004 | 0.006 | 0.013 | 0.033 | 0.069 |

Legend: Ratios are sorted in descending order of the median. Values are shown to include three decimal figures. Abbreviations as listed in the legend of supplemental Table 1.

**Table S4.** Unadjusted extended percentiles of disease ranges for conditions **CH TSH** and **FP TSH.**

| | Marker | 1%ile | 5%ile | 10%ile | 25%ile | 50%ile | 75%ile | 90%ile | 99%ile |
|---|---|---|---|---|---|---|---|---|---|
| **CH TSH** | | | | | | | | | |
| HIGH | TSH | 23.5 | 26.2 | 28.8 | 35.5 | 76 | 243 | 400 | 777 |
| HIGH | TSH/BIOT A | 0.43 | 0.51 | 0.63 | 0.83 | 1.68 | 4.96 | 7.63 | 13.63 |
| HIGH | TSH/BIOT F% | 0.27 | 0.33 | 0.39 | 0.55 | 1.97 | 4.33 | 6.17 | 10.12 |
| HIGH | TSH/IRT | 0.46 | 0.7 | 0.96 | 1.47 | 3.08 | 10.25 | 20.27 | 44.9 |
| HIGH | TSH/CIT | 1.26 | 1.49 | 1.81 | 2.54 | 5.29 | 15.14 | 24.72 | 72.5 |
| HIGH | TSH/TYR | 0.18 | 0.27 | 0.32 | 0.47 | 0.99 | 2.6 | 4.49 | 9.75 |
| HIGH | TSH/GALT | 0.065 | 0.086 | 0.11 | 0.14 | 0.35 | 0.96 | 1.38 | 2.31 |
| HIGH | TSH/GALT% | 0.26 | 0.31 | 0.32 | 0.46 | 1.54 | 4.56 | 6.61 | 12.51 |
| HIGH | TSH/TRECs | 0.01 | 0.016 | 0.027 | 0.14 | 0.45 | 1.29 | 2.39 | 5.58 |
| LOW | 17OHP/TSH | 0.0075 | 0.015 | 0.022 | 0.046 | 0.1 | 0.23 | 0.39 | 0.87 |
| LOW | C3/TSH | 0.0019 | 0.0028 | 0.0038 | 0.0069 | 0.02 | 0.043 | 0.068 | 0.13 |
| LOW | C16/TSH | 0.0026 | 0.0042 | 0.0059 | 0.011 | 0.034 | 0.075 | 0.11 | 0.19 |
| LOW | GALC/TSH | 0.011 | 0.016 | 0.018 | 0.043 | 0.1 | 0.15 | 0.26 | 0.36 |
| **FP TSH** | | | | | | | | | |
| HIGH | TSH | 24.69 | 25.4 | 25.8 | 27.7 | 31.7 | 42.3 | 66.7 | 113 |
| HIGH | TSH/BIOT A | 0.36 | 0.43 | 0.47 | 0.56 | 0.71 | 1.07 | 1.62 | 3.00 |
| HIGH | TSH/BIOT F% | 0.19 | 0.22 | 0.23 | 0.27 | 0.34 | 0.39 | 0.51 | 0.67 |
| HIGH | TSH/IRT | 0.29 | 0.48 | 0.59 | 0.84 | 1.26 | 1.93 | 2.8 | 6.2 |
| HIGH | TSH/CIT | 0.87 | 1.18 | 1.37 | 1.74 | 2.37 | 3.42 | 4.77 | 9.62 |
| HIGH | TSH/TYR | 0.16 | 0.21 | 0.25 | 0.32 | 0.44 | 0.67 | 0.99 | 1.79 |
| HIGH | TSH/GALT | 0.059 | 0.074 | 0.083 | 0.1 | 0.13 | 0.21 | 0.33 | 0.66 |
| HIGH | TSH/GALT% | 0.25 | 0.26 | 0.27 | 0.31 | 0.33 | 0.39 | 0.47 | 0.56 |
| HIGH | TSH/TRECs | 0.0087 | 0.011 | 0.013 | 0.023 | 0.054 | 0.11 | 0.23 | 0.51 |
| LOW | 17OHP/TSH | 0.073 | 0.12 | 0.15 | 0.21 | 0.3 | 0.44 | 0.62 | 1.14 |
| LOW | C3/TSH | 0.0096 | 0.015 | 0.02 | 0.03 | 0.045 | 0.064 | 0.085 | 0.14 |
| LOW | C16/TSH | 0.012 | 0.02 | 0.026 | 0.05 | 0.08 | 0.11 | 0.13 | 0.20 |
| LOW | GALC/TSH | 0.025 | 0.038 | 0.052 | 0.076 | 0.12 | 0.18 | 0.27 | 0.59 |

**Figure S1.** Plot by Multiple Conditions comparing disease ranges of conditions **CH TSH** and **FP TSH.**

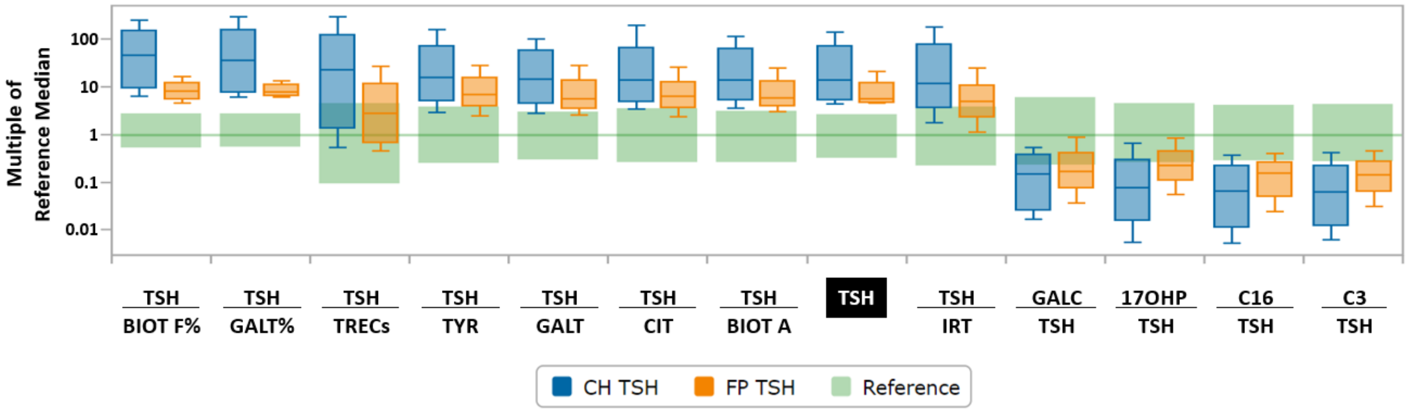

**Table S5.** Unadjusted extended percentiles of disease ranges for conditions **CH T4** and **FP T4**.

| | Marker | 1%ile | 5%ile | 10%ile | 25%ile | 50%ile | 75%ile | 90%ile | 99%ile |
|---|---|---|---|---|---|---|---|---|---|
| **CH T4** | | | | | | | | | |
| HIGH | TSH/T4 | 0.30 | 0.38 | 0.52 | 0.74 | 1.35 | 2.74 | 4.74 | 10.09 |
| HIGH | 17OHP/T4 | 0.26 | 0.30 | 0.43 | 1.47 | 3.84 | 10.40 | 20.09 | 49.6 |
| HIGH | C3/T4 | 0.072 | 0.094 | 0.11 | 0.19 | 0.38 | 0.64 | 1.13 | 1.55 |
| HIGH | C16/T4 | 0.05 | 0.08 | 0.13 | 0.18 | 0.33 | 0.54 | 0.89 | 1.51 |
| HIGH | CIT/T4 | 0.99 | 1.57 | 2.00 | 2.81 | 4.11 | 6.55 | 12.47 | 20.14 |
| HIGH | GALC/T4 | 0.36 | 0.41 | 0.51 | 0.69 | 1.46 | 4.18 | 5.43 | 8.82 |
| LOW | T4 | 1.07 | 1.14 | 1.58 | 2.21 | 3.98 | 5.22 | 6.97 | 8.01 |
| LOW | T4/BIOT A | 0.021 | 0.026 | 0.029 | 0.064 | 0.110 | 0.130 | 0.190 | 0.26 |
| LOW | T4/GALT | 0.0038 | 0.0045 | 0.0065 | 0.012 | 0.020 | 0.031 | 0.047 | 0.077 |
| LOW | T4/IRT | 0.011 | 0.028 | 0.037 | 0.083 | 0.170 | 0.320 | 0.410 | 0.71 |
| LOW | T4/TRECs | 0.0011 | 0.0016 | 0.0026 | 0.0034 | 0.0058 | 0.011 | 0.017 | 0.025 |
| LOW | T4/TYR | 0.006 | 0.008 | 0.009 | 0.025 | 0.047 | 0.081 | 0.110 | 0.25 |
| **FP T4** | | | | | | | | | |
| HIGH | TSH/T4 | 0.20 | 0.29 | 0.34 | 0.51 | 0.86 | 1.5 | 2.31 | 4.57 |
| HIGH | 17OHP/T4 | 0.24 | 0.52 | 0.83 | 1.73 | 4.08 | 9.21 | 17.29 | 44.30 |
| HIGH | C3/T4 | 0.053 | 0.089 | 0.12 | 0.19 | 0.30 | 0.48 | 0.73 | 1.41 |
| HIGH | C16/T4 | 0.054 | 0.09 | 0.12 | 0.18 | 0.27 | 0.4 | 0.56 | 1.07 |
| HIGH | CIT/T4 | 0.95 | 1.37 | 1.63 | 2.35 | 3.67 | 5.58 | 8.20 | 17.18 |
| HIGH | GALC/T4 | 0.20 | 0.35 | 0.49 | 0.87 | 1.89 | 3.72 | 6.19 | 12.88 |
| LOW | T4 | 1.15 | 1.69 | 2.22 | 3.23 | 4.29 | 5.40 | 7.16 | 8.12 |
| LOW | T4/BIOT A | 0.029 | 0.047 | 0.059 | 0.083 | 0.12 | 0.17 | 0.24 | 0.41 |
| LOW | T4/GALT | 0.0063 | 0.01 | 0.013 | 0.018 | 0.025 | 0.036 | 0.048 | 0.079 |
| LOW | T4/IRT | 0.02 | 0.037 | 0.058 | 0.1 | 0.19 | 0.31 | 0.48 | 1 |
| LOW | T4/TRECs | 0.00068 | 0.0014 | 0.0018 | 0.003 | 0.0055 | 0.01 | 0.019 | 0.068 |
| LOW | T4/TYR | 0.0072 | 0.014 | 0.021 | 0.034 | 0.053 | 0.08 | 0.12 | 0.23 |

**Supplemental Figure S2.** Plot by Multiple Conditions comparing disease ranges of conditions **CH T4** and **FP T4**.

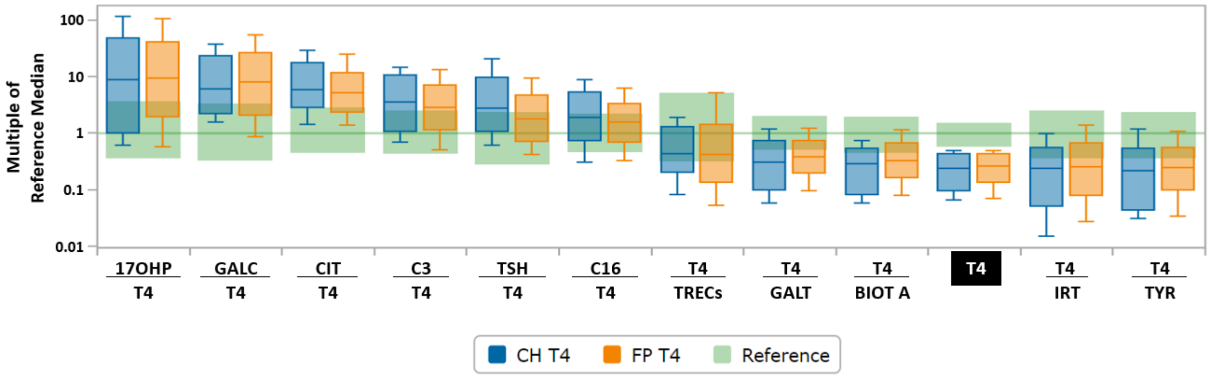

**Table S6.** Unadjusted extended percentiles of disease ranges for conditions **CH TSH T4** and **FP TSH T4**

| | Marker | 1%ile | 5%ile | 10%ile | 25%ile | 50%ile | 75%ile | 90%ile | 99%ile |
|---|---|---|---|---|---|---|---|---|---|
| **CH TSH T4** | | | | | | | | | |
| HIGH | TSH | 15.06 | 20.66 | 30.9 | 75.4 | 276 | 534 | 687 | 1,180 |
| HIGH | TSH/T4 | 2.38 | 3.37 | 4.9 | 14.03 | 56.8 | 159 | 294 | 664 |
| HIGH | TSH/BIOT A | 0.32 | 0.47 | 0.55 | 1.39 | 4.6 | 13.51 | 19.64 | 37 |
| HIGH | TSH/GALT | 0.049 | 0.089 | 0.13 | 0.29 | 1.15 | 2.36 | 3.82 | 8.75 |
| HIGH | TSH/CIT | 0.84 | 1.42 | 2.09 | 5.44 | 20.25 | 39.5 | 59.6 | 122 |
| HIGH | TSH/TYR | 0.12 | 0.25 | 0.42 | 1.12 | 3.05 | 6.14 | 9.08 | 18.4 |
| HIGH | TSH/IRT | 0.19 | 0.76 | 1.16 | 3.59 | 13.54 | 30.1 | 45.8 | 102 |
| HIGH | TSH/TRECs | 0.0087 | 0.014 | 0.021 | 0.045 | 0.18 | 0.4 | 1 | 2.5 |
| HIGH | 17OHP/T4 | 0.22 | 0.47 | 0.66 | 1.14 | 2.16 | 4.4 | 9.94 | 36.6 |
| HIGH | C3/T4 | 0.043 | 0.096 | 0.13 | 0.2 | 0.32 | 0.6 | 0.9 | 1.65 |
| HIGH | C16/T4 | 0.068 | 0.11 | 0.16 | 0.28 | 0.44 | 0.72 | 1.11 | 2.24 |
| HIGH | CIT/T4 | 0.75 | 1.01 | 1.21 | 1.79 | 2.93 | 5.24 | 8.68 | 23.72 |
| HIGH | GALC/T4 | 0.21 | 0.29 | 0.37 | 0.59 | 0.92 | 2.15 | 4.17 | 8.34 |
| LOW | 17OHP/TSH | 0.0043 | 0.0095 | 0.012 | 0.02 | 0.037 | 0.11 | 0.33 | 1.53 |
| LOW | C3/TSH | 0.0007 | 0.0014 | 0.0017 | 0.0027 | 0.0055 | 0.017 | 0.051 | 0.11 |
| LOW | C16/TSH | 0.0012 | 0.0017 | 0.0022 | 0.004 | 0.0074 | 0.022 | 0.048 | 0.14 |
| LOW | GALC/TSH | 0.0029 | 0.0035 | 0.0053 | 0.01 | 0.032 | 0.11 | 0.28 | 0.89 |
| LOW | T4 | 1.02 | 1.42 | 1.84 | 2.83 | 4.62 | 6.92 | 8.26 | 9.29 |
| LOW | T4/BIOT A | 0.024 | 0.033 | 0.042 | 0.064 | 0.11 | 0.14 | 0.2 | 0.26 |
| LOW | T4/GALT | 0.0038 | 0.0056 | 0.0075 | 0.012 | 0.022 | 0.034 | 0.047 | 0.073 |
| LOW | T4/IRT | 0.021 | 0.049 | 0.074 | 0.13 | 0.22 | 0.37 | 0.51 | 0.94 |
| LOW | T4/TRECs | 0.00086 | 0.0015 | 0.0018 | 0.0027 | 0.0047 | 0.0075 | 0.015 | 0.034 |
| LOW | T4/TYR | 0.0095 | 0.014 | 0.018 | 0.03 | 0.057 | 0.092 | 0.13 | 0.25 |
| **FP TSH T4** | | | | | | | | | |
| HIGH | TSH | 14.53 | 15.94 | 18.12 | 26.2 | 43.9 | 63.2 | 81.1 | 140 |
| HIGH | TSH/T4 | 2.04 | 2.7 | 3.17 | 4.45 | 6.69 | 9.11 | 11.93 | 22.84 |
| HIGH | TSH/BIOT A | 0.3 | 0.4 | 0.48 | 0.71 | 1.17 | 1.63 | 2.31 | 4.6 |
| HIGH | TSH/GALT | 0.045 | 0.063 | 0.081 | 0.12 | 0.2 | 0.29 | 0.42 | 0.9 |
| HIGH | TSH/CIT | 0.69 | 0.98 | 1.2 | 1.91 | 3.14 | 4.53 | 6.2 | 12.1 |
| HIGH | TSH/TYR | 0.12 | 0.17 | 0.22 | 0.34 | 0.61 | 0.92 | 1.27 | 2.25 |
| HIGH | TSH/IRT | 0.087 | 0.22 | 0.35 | 0.71 | 1.41 | 2.77 | 4.44 | 9.73 |
| HIGH | TSH/TRECs | 0.0057 | 0.011 | 0.012 | 0.018 | 0.026 | 0.047 | 0.081 | 0.5 |
| HIGH | 17OHP/T4 | 0.49 | 0.89 | 1.2 | 1.96 | 3.19 | 5.2 | 7.89 | 18.69 |
| HIGH | C3/T4 | 0.072 | 0.1 | 0.12 | 0.16 | 0.22 | 0.32 | 0.43 | 0.79 |
| HIGH | C16/T4 | 0.098 | 0.13 | 0.15 | 0.2 | 0.27 | 0.38 | 0.49 | 0.77 |
| HIGH | CIT/T4 | 0.74 | 1.05 | 1.24 | 1.65 | 2.14 | 2.93 | 3.92 | 7.43 |
| HIGH | GALC/T4 | 0.23 | 0.3 | 0.35 | 0.58 | 1.04 | 2.32 | 4.13 | 9.68 |
| LOW | 17OHP/TSH | 0.066 | 0.13 | 0.17 | 0.27 | 0.47 | 0.89 | 1.5 | 3.55 |
| LOW | C3/TSH | 0.0071 | 0.011 | 0.015 | 0.022 | 0.034 | 0.059 | 0.089 | 0.17 |
| LOW | C16/TSH | 0.0085 | 0.014 | 0.019 | 0.026 | 0.041 | 0.067 | 0.11 | 0.2 |
| LOW | GALC/TSH | 0.026 | 0.05 | 0.066 | 0.14 | 0.23 | 0.48 | 0.94 | 1.88 |
| LOW | T4 | 2.36 | 3.51 | 4.09 | 5.33 | 7.07 | 8.09 | 8.89 | 9.4 |
| LOW | T4/BIOT A | 0.071 | 0.088 | 0.1 | 0.13 | 0.17 | 0.21 | 0.25 | 0.41 |
| LOW | T4/GALT | 0.011 | 0.015 | 0.017 | 0.022 | 0.029 | 0.037 | 0.048 | 0.083 |
| LOW | T4/IRT | 0.016 | 0.035 | 0.059 | 0.13 | 0.23 | 0.37 | 0.56 | 1.06 |
| LOW | T4/TRECs | 0.0011 | 0.0019 | 0.0024 | 0.0041 | 0.006 | 0.01 | 0.019 | 0.05 |
| LOW | T4/TYR | 0.026 | 0.038 | 0.047 | 0.068 | 0.091 | 0.12 | 0.14 | 0.22 |

**Figure S3.** Plot by Multiple Conditions comparing disease ranges of conditions **CH TSH T4** and **FP TSH T4**.

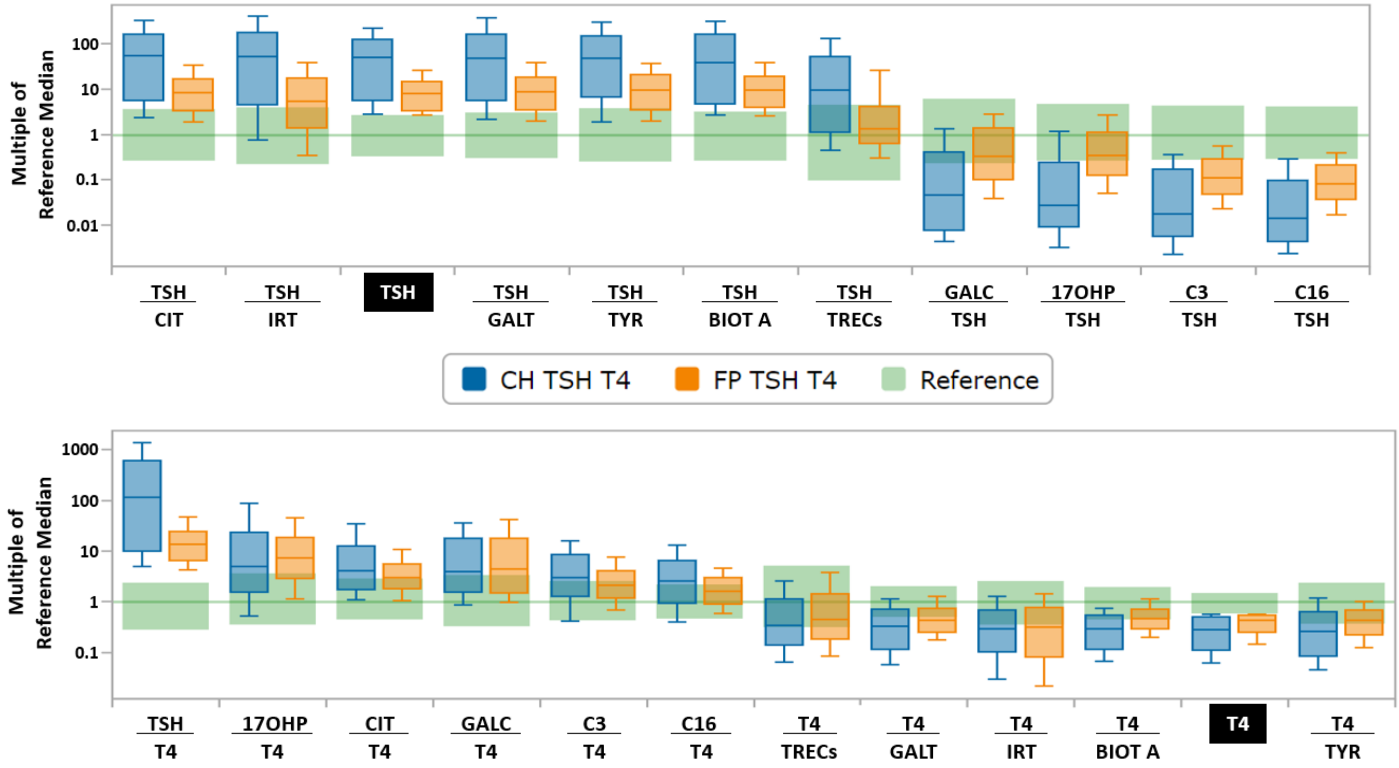

**Figure S4.** Single Condition Tool for condition CH TSH for location California.

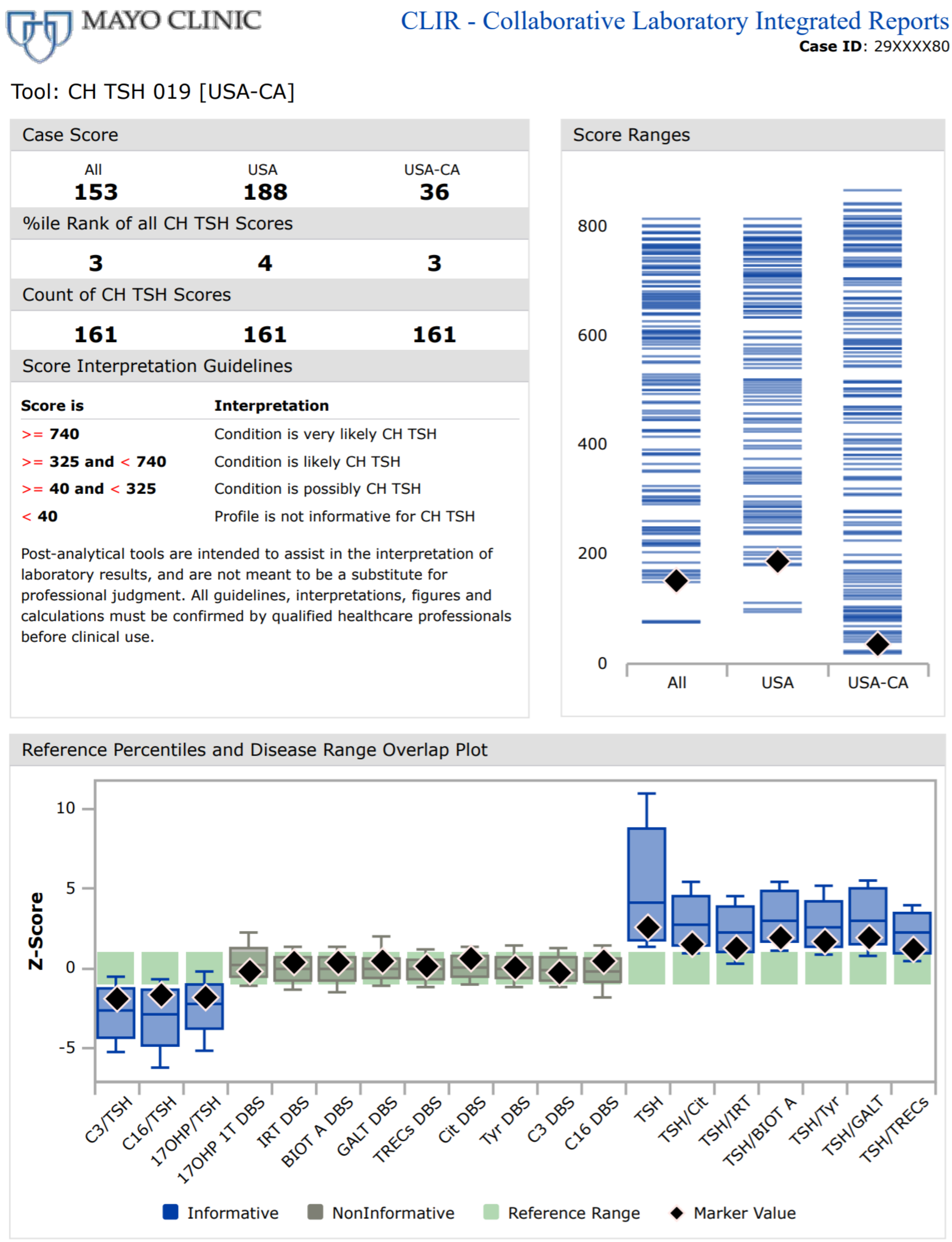

(continued, Figure S4)

### Reference Percentiles and Disease Range Overlap Values

| Markers | | RR | Overlap | Informative Disease Range Scoring %iles | | | | | Case | Adjusted |
|---|---|---|---|---|---|---|---|---|---|---|
| **Low** | **Unit** | **10%ile** | **%ile** | **99** | **95** | **90** | **85** | **50** | **Value** | **Value** |
| C3/TSH | Ratio | -1.3 | 1.0 | -1.3 | -2.27 | -2.89 | -3.2 | -5.89 | 0.05 | -4.16 |
| C16/TSH | Ratio | -1.31 | 0.5 | -1.59 | -2.48 | -3 | -3.47 | -6.53 | 0.12 | -3.66 |
| 17OHP/TSH | Ratio | -1.32 | 3.1 | -0.45 | -1.58 | -2.44 | -2.74 | -5.16 | 0.3 | -4.28 |
| **High** | **Unit** | **90%ile** | **%ile** | **1** | **5** | **10** | **15** | **50** | **Case** | **Adjusted** |
| TSH | m[IU]/L | 0.99 | 0.0 | 2.52 | 2.96 | 3.37 | 3.8 | 8.38 | 32.3 | 5.18 |
| TSH/Cit | Ratio | 1.31 | 0.2 | 2.09 | 2.57 | 3.28 | 3.53 | 6.16 | 1.35 | 3.55 |
| TSH/IRT | Ratio | 1.28 | 3.7 | 0.62 | 1.58 | 2.13 | 2.53 | 4.88 | 1.01 | 2.85 |
| TSH/BIOT A | Ratio | 1.28 | 0.0 | 2.26 | 2.96 | 3.5 | 3.81 | 6.53 | 0.56 | 4.2 |
| TSH/Tyr | Ratio | 1.31 | 0.2 | 1.92 | 2.43 | 3 | 3.37 | 5.78 | 0.34 | 3.86 |
| TSH/GALT | Ratio | 1.3 | 0.6 | 1.59 | 2.56 | 3.22 | 3.69 | 6.55 | 0.1 | 4.31 |
| TSH/TRECs | Ratio | 1.35 | 3.0 | 1.03 | 1.75 | 2.09 | 2.5 | 5.31 | 0.11 | 2.91 |
| | | **RR** | **RR** | **Differentiator Disease Range %iles** | | | | | | |
| **Differentiator** | **Unit** | **10%ile** | **90%ile** | **1** | **10** | **50** | **90** | **99** | **Case** | **Adjusted** |
| 17OHP 1T DBS | ng/mL | -1.3 | 1.35 | -2.37 | -1.19 | 0.54 | 3.08 | 5.49 | 9.55 | -0.38 |
| IRT DBS | ug/L | -1.27 | 1.43 | -2.68 | -1.58 | -0.075 | 1.61 | 2.95 | 31.9 | 0.93 |
| BIOT A DBS | ERU | -1.44 | 1.26 | -3.62 | -1.92 | -0.03 | 1.37 | 2.62 | 57.5 | 0.82 |
| GALT DBS | U/g[Hb] | -1.35 | 1.27 | -2.55 | -1.5 | -0.083 | 1.55 | 4.78 | 310 | 1.19 |
| TRECs DBS | copies/uL | -1.44 | 1.22 | -2.94 | -1.85 | -0.096 | 1.16 | 2.48 | 302 | 0.36 |
| Cit DBS | nmol/mL | -1.34 | 1.33 | -2.4 | -1.25 | 0.21 | 1.68 | 3.08 | 24 | 1.51 |
| Tyr DBS | nmol/mL | -1.33 | 1.39 | -2.57 | -1.41 | -0.031 | 1.6 | 3.2 | 94.2 | 0.16 |
| C3 DBS | nmol/mL | -1.32 | 1.37 | -2.6 | -1.75 | -0.18 | 1.55 | 2.84 | 1.6 | -0.53 |
| C16 DBS | nmol/mL | -1.38 | 1.31 | -4.24 | -2.09 | -0.37 | 1.29 | 2.95 | 3.99 | 1.02 |

RR - DR Overlap

| Covariate | Value |
|---|---|
| Age hr | 28 |
| BW | 4030 |
| Sex | Male |

**Figure S5.** Reference range comparison of unadjusted and adjusted values for marker **TRECs**.

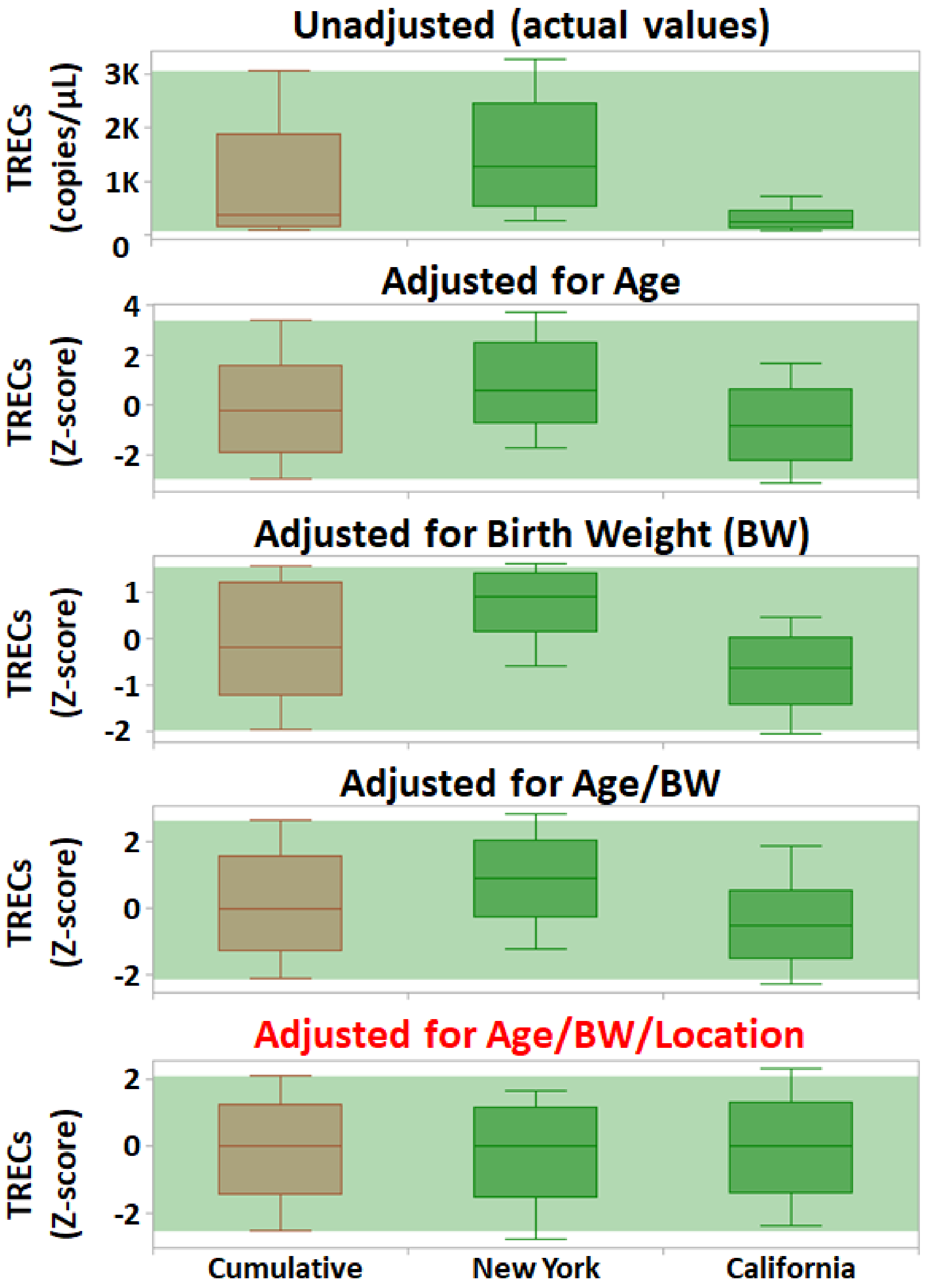


Legend: New York N=353,322; California N=520,539.


© 2021 by the authors. Submitted for possible open access publication under the terms and conditions of the Creative Commons Attribution (CC BY) license (http://creativecommons.org/licenses/by/4.0/).
